# Safety and antitumor activity of copanlisib in Japanese patients with relapsed/refractory indolent non-Hodgkin lymphoma: a phase Ib/II study

**DOI:** 10.1007/s12185-022-03455-0

**Published:** 2022-09-29

**Authors:** Noriko Fukuhara, Dai Maruyama, Kiyohiko Hatake, Hirokazu Nagai, Shinichi Makita, Kenjiro Kamezaki, Toshiki Uchida, Shigeru Kusumoto, Junya Kuroda, Chisako Iriyama, Masamitsu Yanada, Norifumi Tsukamoto, Youko Suehiro, Hironobu Minami, Jose Garcia-Vargas, Barrett H. Childs, Masanobu Yasuda, Shigeo Masuda, Toshiaki Tsujino, Yui Terao, Kensei Tobinai

**Affiliations:** 1 Department of Hematology, Tohoku University Hospital, Sendai, 980-8574 Japan; 2 Department of Hematology, National Cancer Center Hospital, Tokyo, Japan; 3 Department of Hematology Oncology, Cancer Institute Hospital, Japanese Foundation for Cancer Research, Tokyo, Japan; 4 Department of Hematology, National Hospital Organization Nagoya Medical Center, Nagoya, Japan; 5 Center for Cellular and Molecular Medicine, Kyushu University Hospital, Fukuoka, Japan; 6 Department of Hematology and Oncology, Japanese Red Cross Nagoya Daini Hospital, Nagoya, Japan; 7 Department of Hematology and Oncology, Nagoya City University Graduate School of Medical Sciences, Nagoya, Japan; 8 Division of Hematology and Oncology, Kyoto Prefectural University of Medicine, Kyoto, Japan; 9 Department of Hematology and Cell Therapy, Aichi Cancer Center, Nagoya, Japan; 10 Department of Medicine and Clinical Science, Gunma University Hospital, Gunma, Japan; 11 Department of Hematology, National Hospital Organization Kyushu Cancer Center, Fukuoka, Japan; 12 Department of Medical Oncology/Hematology, Kobe University Hospital, Kobe, Japan; 13 Bayer HealthCare Pharmaceuticals, Inc, Whippany, NJ USA; 14 Bayer Yakuhin, Ltd, Osaka, Japan

**Keywords:** Copanlisib, Hyperglycemia, Japanese, Non-Hodgkin lymphoma

## Abstract

The safety, efficacy, and pharmacokinetics of copanlisib were evaluated in this phase Ib/II study in Japanese patients with relapsed/refractory indolent non-Hodgkin lymphoma (NHL). The primary endpoint was safety at the recommended dose; efficacy endpoints included objective response rate (ORR), progression-free survival (PFS), and overall survival. In phase Ib, patients received copanlisib 45 mg intravenously on days 1, 8, and 15 of a 28-day cycle, and when tolerated, consecutive patients received copanlisib 60 mg. As no dose-limiting toxicities occurred at the 45 mg (*n* = 3) or 60 mg (*n* = 7) dose in phase Ib, the recommended dose for Japanese patients was determined to be 60 mg, and this dose was used in phase II (*n* = 15). Although all patients experienced at least one treatment-emergent adverse event (TEAE), with hyperglycemia being the most common AE, no AE-related deaths were reported. The ORR was 68.0% (17/25 patients), median PFS was 302 (95% CI 231–484) days, and the duration of response was 330 (range 65–659) days. The pharmacokinetic properties of copanlisib were similar between Japanese and non-Japanese patients. Overall, copanlisib 60 mg had an acceptable safety profile and showed promising antitumor activity in Japanese patients with relapsed/refractory indolent NHL.

**Supplementary Information:**

The online version contains supplementary material available at 10.1007/s12185-022-03455-0.

## Introduction

Non-Hodgkin lymphomas (NHLs) comprise about 95% of the malignant lymphomas diagnosed in Japan [1]. While the overall prevalence of NHL is lower in Asian countries compared with Western countries, the prevalence of NHL is growing in Japan at a faster rate than in the United States of America (USA) [2]. Although indolent NHL is slow-growing, more than 90% of patients with indolent NHL have advanced disease at presentation, and advanced indolent NHL is incurable and requires treatment [3]. Recommended first- and second-line treatments are rituximab-based chemotherapy regimens (usually R-CHOP [i.e., rituximab, cyclophosphamide, hydroxydaunorubicin hydrochloride (doxorubicin hydrochloride), vincristine, and prednisone] or rituximab + bendamustine) [4–6], but treatment options after second-line therapy are limited.

Phosphatidylinositol 3 kinases (PI3K) are a family of membrane-bound kinases involved in the growth and survival of healthy cells that are implicated in tumor growth and proliferation when they are dysregulated or aberrantly activated [7]. Copanlisib is a pan-PI3K inhibitor, with potent activity against the p110α and p110δ isoforms [8]. Copanlisib is approved and recommended in the USA for patients with relapsed or refractory follicular lymphoma (FL) or marginal zone lymphoma (MZL) who have received at least two prior therapies [4, 9]. In a previous pharmacokinetic analysis of copanlisib in Japanese patients with advanced or refractory solid tumors [10], copanlisib administered at 0.4 or 0.8 mg/kg (approximately equivalent to a 60-mg flat dose) showed similar pharmacokinetic exposure to that observed in the first-in-human study in non-Japanese patients with solid tumors or NHL [11].

This article describes a phase Ib/II study in Japanese patients with relapsed/refractory indolent NHL. The aims of the study were to identify the recommended dose of copanlisib in this patient group and assess the safety and antitumor activity of the copanlisib recommended dose.

## Methods

### Study design

This was an open-label, single-arm, phase Ib/II study conducted at 13 centers in Japan. The study was in two parts: the dose-escalation part (phase Ib) to determine the recommended dose and the expansion part (phase II) to evaluate the antitumor activity and safety of the recommended dose of copanlisib.

The protocol was reviewed and approved by each study site’s Independent Ethics Committee/Institutional Review Board before the start of the study, as were all protocol amendments at the time of their implementation. The study was conducted in accordance with the ethical principles that have their origin in the Declaration of Helsinki and the International Council for Harmonisation guideline E6: Good Clinical Practice. All patients voluntarily provided written informed consent prior to any study procedures being undertaken.

### Patients

The study included Japanese patients aged ≥ 20 years with a histologically confirmed diagnosis (central pathology review) of one of the following types of indolent B-cell NHL: FL grade 1, 2, or 3a, small lymphocytic lymphoma with absolute lymphocyte count < 5 × 10^9^/L at the time of diagnosis and at study entry, lymphoplasmacytic lymphoma/Waldenström macroglobulinemia (LPL/WM), or MZL (splenic, nodal, or extra-nodal). Only patients with NHL that was relapsed or refractory after at least two prior lines of therapy (refractory was defined as not responding to a standard regimen or progressing within 6 months of the last course of a standard regimen) were included. Prior therapy must have included rituximab and an alkylating agent.

Other inclusion criteria were: Eastern Cooperative Oncology Group (ECOG) performance status 0–2, life expectancy of 3 months or longer, adequate bone marrow, liver, and renal function as assessed within 7 days before starting study treatment, left ventricular ejection fraction ≥ lower limit of normal for the study center, and the availability of fresh or archival tumor tissue.

Key exclusion criteria were: uncontrolled hypertension (blood pressure > 150/90 mmHg despite optimal medical management), type I or II diabetes mellitus (glycated hemoglobin > 8.5% or fasting blood glucose > 160 mg/dL at screening), history of cardiac disease or congestive heart failure, myocardial infarction within 6 months of study entry, active heart disease, new-onset angina within 3 months before study entry, treatment (chemotherapy, immunotherapy, radiotherapy, or investigational drug) targeting lesions within 4 weeks before study entry, evidence or history of a bleeding diathesis, history or concurrent conditions or interstitial lung disease or impaired pulmonary function, ongoing systemic corticosteroid therapy, unresolved toxicity attributed to prior treatment (other than alopecia), prior treatment with a PI3K inhibitor or strong cytochrome P450 3A4 inhibitors, known history of human immunodeficiency virus infection or chronic hepatitis B or C virus infection requiring treatment, seizure disorders, inadequate liver, renal, or bone marrow function (assessed as per laboratory values), and pregnant or lactating women.

### Study methodology

After a screening period of up to 28 days, three eligible patients began treatment with copanlisib at 45 mg (dose level 1) by 1 h intravenous infusion on days 1, 8, and 15 of a 28-day cycle. Tolerability was assessed at the end of the first cycle, and if no dose-limiting toxicities (DLTs) occurred, three additional patients were enrolled and treated with copanlisib at 60 mg (dose level 2). If one DLT developed, an additional three patients were enrolled and treated at each dose level. Copanlisib was considered not to be tolerated and the study stopped if two or more out of three patients or two or more out of six patients showed DLTs.

Once the recommended dose level was established, 20 patients were enrolled in the expansion part (phase II) of the study, which included patients from the dose-escalation phase. During the expansion part, patients were treated with copanlisib until disease progression (PD), clinical progression (e.g., deterioration of ECOG performance status to ≥ 3), unacceptable toxicity, or other reasons for treatment withdrawal.

As transient post-infusion hyperglycemia is a known adverse event (AE) with copanlisib, a low carbohydrate diet was recommended for the first 48 h after copanlisib infusion.

### Outcomes and assessments

Patients were assessed for AEs, and blood samples were taken for laboratory assessments at screening, on day 1 of cycle 1, at each visit during treatment, and at the safety follow-up visit 28–35 days after the last administration of copanlisib. AEs were graded using the National Cancer Institute Common Terminology Criteria for Adverse Events (version 4), and assessed as related or not related to copanlisib by the investigator. Treatment-emergent AEs (TEAEs) were any AEs arising or worsening after the start of drug administration until 28–35 days after the last dose of copanlisib, and an adverse drug reaction (ADR) was an AE considered to be related to copanlisib treatment.

A DLT was defined as any grade 3 or 4 non-hematologic toxicity that did not improve within 21 days despite optimal supportive care, excluding the following events: transient post-infusion grade 3 hypertension, grade 3 or 4 hyperglycemia that improves with insulin treatment, and any grade 3 or 4 lipase and/or amylase elevation without signs of pancreatitis. Hematologic DLTs were defined as grade 4 neutropenia lasting for ≥ 7 days despite adequate treatment (including granulocyte colony stimulating factor), an absolute neutrophil count < 1000/mm^3^ with fever ≥ 38.5 °C, grade 4 thrombocytopenia or grade 3 thrombocytopenia associated with serious bleeding, grade 4 anemia, and signs of serious bleeding and/or an international normalized ratio of > 2.5 times the upper limit of normal (ULN) and/or activated partial thromboplastin time elevation of 2.5 × ULN. Pre-specified dose modifications were implemented in patients who developed a DLT.

The primary endpoint was to assess the safety profile of copanlisib at the recommended dose. Contrast-enhanced computed tomography or magnetic resonance imaging of all suspected disease sites was performed at screening, every 2 cycle during study treatment during Year 1, every 3 cycle during Year 2, and every 6 cycle during Year 3. An independent, blinded evaluation of the tumor status was conducted using the Revised Response Criteria for Malignant Lymphoma [12]. The primary efficacy endpoint was objective response rate (ORR), i.e., number of patients with a best response of complete response (CR) or partial response (PR). Progression-free survival (PFS) was defined as the time from the first administration of copanlisib to the first disease progression (radiologic or clinical PD or first AE associated with clinical PD, whichever was earlier) or death from any cause. Overall survival (OS) was defined as the time from the first administration of copanlisib to death from any cause. A pharmacokinetic analysis was undertaken after cycle 1 in the dose-escalation and expansion parts (Methodology described in supplementary materials).

### Statistical analysis

Since the study was primarily a descriptive safety and tolerability assessment, no formal sample size estimate was performed, but we included at least three patients at each dose level to identify any DLTs and 20 patients to determine antitumor activity. Both efficacy and safety were assessed in the full analysis set (FAS), which included all patients who were assigned to treatment in the dose-escalation part (FAS 1) or dose-expansion part (FAS 2). Demographics and baseline characteristics were summarized using descriptive statistics and/or frequency as appropriate. Efficacy variables were summarized using descriptive statistics, with 95% confidence intervals (CIs) calculated for ORR. Time-to-event endpoints were assessed using frequency, median, quartiles, range, and rate within each 90-day period, as well as by Kaplan–Meier curves. Missing data were not imputed.

## Results

### Patients

Thirty-two Japanese patients were enrolled in this study, and the FAS included 25 patients. Ten patients were included in FAS 1 (i.e., the phase Ib dose-escalation cohort: three patients in the 45 mg cohort and seven in the 60 mg cohort), and 15 in FAS 2 (i.e., the expansion cohort). FAS 1 included one patient who received copanlisib 60 mg but did not have a blood glucose measurement taken within 24 h of the infusion on day 8 of cycle 1, so could not be evaluated for hyperglycemia development and DLTs. This patient was replaced with another for DLT assessment in FAS 1, so that six patients could be evaluated for DLTs.

**Fig. 1:**
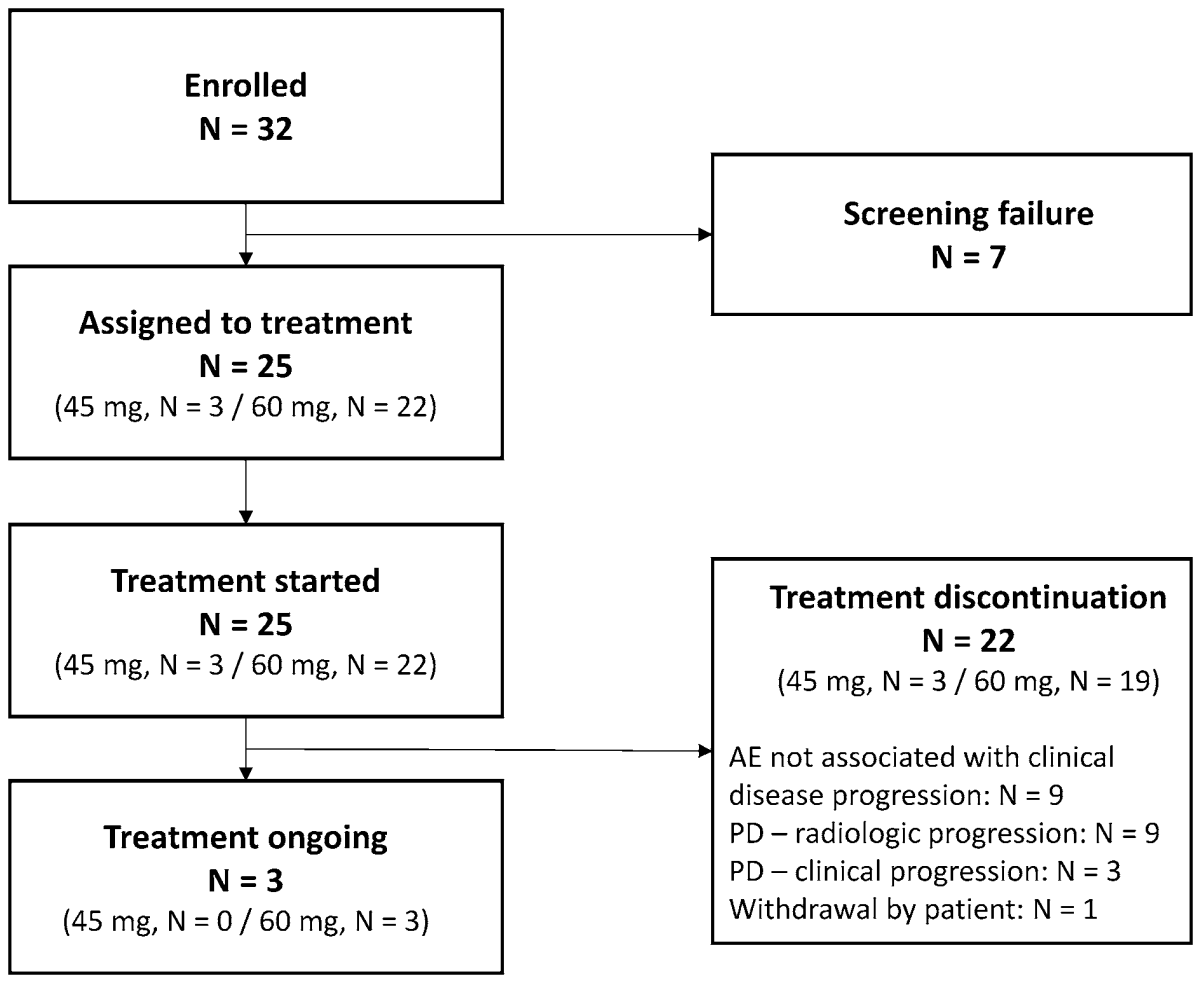
Study flow chart. The data cut-off date was February 20, 2020. *AE* adverse event, *PD* progressive disease

At the time of data cut-off, three patients continued to receive copanlisib, 22 had discontinued, primarily because of AEs (*n* = 9) and radiologic disease progression (*n* = 9) (Fig. 1), and six patients had died.

Thirteen patients were male (52.0%) and 12 were female (48.0%), their median age was 67 years, and 16/25 patients (64.0%) were aged ≥ 65 years (Table 1). The most common diagnosis was FL (*n* = 23; 92.0%). Of the 23 patients with lymphoma grade information available at baseline, tumor grade was 1 in six patients, 2 in 11 patients, and 3a in six patients. Two of the 23 patients with investigator-assessed FL were later identified as not FL through histology assessment. Most patients (*n* = 19; 76.0%) had a tumor diameter of < 7 cm (mean 5.3 cm). All patients had previously received systemic chemotherapy, including rituximab and alkylating agents, and three had received radiotherapy (Table 2). The number of prior lines of anticancer chemotherapy ranged from 2 to 13 (median 3).

**Table 1:** Baseline demographic and clinical characteristics

	Copanlisib		
	45 mg (*n* = 3)	60 mg (*n* = 22)	Total (*n* = 25)
Sex, *n* (%)			
Male/Female	1 (33.3)/2 (66.7)	12 (54.5)/10 (45.5)	13 (52.0)/12 (48.0)
Age, years, mean (SD)	69.0 (3.5)	64.3 (8.8)	64.9 (8.4)
Age categories, *n* (%)			
< 65 years / ≥ 65 years	0/3 (100.0)	9 (40.9)/13 (59.1)	9 (36.0)/16 (64.0)
Weight, kg, mean (SD)	57.1 (18.8)	61.1 (10.8)	60.6 (11.6)
Blood pressure, mean (SD)			
Systolic / Diastolic	122.7 (11.0)/68.3 (9.6)	123.3 (11.7) / 71.8 (11.4)	123.2 (11.4) / 71.4 (11.1)
ECOG performance status, *n* (%)			
0/1	2 (66.7) / 1 (33.3)	16 (72.7)/6 (27.3)	18 (72.0)/7 (28.0)
Heart failure^a^, *n* (%)	0	1 (4.5)	1 (4.0)
Lymphoma histology^b^, *n* (%)			
Follicular lymphoma	3 (100.0)	20 (90.9)	23 (92.0)
Nodal marginal zone lymphoma	0	1 (4.5)	1 (4.0)
Waldenström macroglobulinemia	0	1 (4.5)	1 (4.0)
Follicular lymphoma grade, *n* (%)			
1	0	6 (27.3)	6 (24.0)
2	3 (100.0)	8 (36.4)	11 (44.0)
3a	0	6 (27.3)	6 (24.0)
Missing	0	2 (9.1)	2 (8.0)
Tumor diameter, cm, mean (SD)	4.0 (1.6)	5.5 (2.1)	5.3 (2.1)
Longest diameter of target lesion, *n* (%)			
< 7 cm / ≥ 7 cm	3 (100.0)/0	16 (72.7)/6 (27.3)	19 (76.0)/6 (24.0)
Time from most recent progression to study entry, weeks, median (range)	7.0 (6–12)	6.0 (2–207)	6.1 (2–207)
Ann Arbor stage, *n* (%)			
I	0	2 (9.1)	2 (8.0)
II	0	5 (22.7)	5 (20.0)
III	2 (66.7)	3 (13.6)	5 (20.0)
IIIS	0	1 (4.5)	1 (4.0)
IV	1 (33.3)	11 (50.0)	12 (48.0)
FLIPI risk category (score), *n* (%)			
Low (0–1)	0	3 (13.6)	3 (12.0)
Intermediate (2)	0	8 (36.4)	8 (32.0)
High (3–5)	3 (100.0)	9 (40.9)	12 (48.0)
Missing	0	2 (9.1)	2 (8.0)

*ECOG* Eastern Cooperative Oncology Group, *FLIPI* Follicular lymphoma international prognostic index, *SD* standard deviation

^a^New York Heart Association class II

^b^Investigator assessed

**Table 2:** Prior anticancer therapies and procedures

Prior treatment^a^, *n* (%)	Copanlisib		
	45 mg (*n* = 3)	60 mg (*n* = 22)	Total (*n* = 25)
Systemic anticancer therapy	3 (100.0)	22 (100.0)	25 (100.0)
Rituximab	3 (100.0)	22 (100.0)	25 (100.0)
Alkylating agents	3 (100.0)	22 (100.0)	25 (100.0)
Number of prior lines of systemic therapy			
< 4	2 (66.7)	11 (50.0)	13 (52.0)
≥ 4	1 (33.3)	11 (50.0)	12 (48.0)
Diagnostic and/or therapeutic procedure	3 (100.0)	21 (95.5)	24 (96.0)
Radiotherapy	0	3 (13.6)	3 (12.0)
High-dose chemotherapy and autologous transplant	0	0	0
Surgical therapeutic procedure	0	0	0

^a^May not include all investigational compounds

### Treatment exposure

At the time of data cut-off, the median duration of study treatment was 19.3 weeks (4.8 cycles), and the median number of copanlisib infusions was 12. The median total dose per patient was 720 mg, and the median percentage of planned dose was 83.3%.

### Safety and tolerability

All patients experienced at least one TEAE (Tables 3, 4). Thirteen patients (52.0%) had a grade 3 AE and 11 (44.0%) had a grade 4 AE. No patient died because of an AE. Serious AEs occurred in 10 patients (40.0%) and serious ADRs occurred in 7 (28.0%). The most common types of AEs with copanlisib were hyperglycemia (*n* = 22; 88.0%), neutrophil count decreased (*n* = 15; 60.0%), white blood cell count decreased (*n* = 14; 56.0%), stomatitis (*n* = 13; 52.0%), and hypertension (*n* = 13; 52.0%) (Table 4). Hyperglycemia was transient, and blood glucose levels returned to normal at the end of the treatment (Supplementary Fig. S1). Hyperglycemia medications were administered as needed to control blood glucose levels (Supplementary Table S1). Similar ADRs were seen with either dose; the overall incidence of ADRs with copanlisib are shown in Table 5.

**Table 3:** Safety

*n* (%)	Copanlisib		
	45 mg (*n* = 3)	60 mg (*n* = 22)	Total (*n* = 25)
Any AE	3 (100.0)	22 (100.0)	25 (100.0)
AE by worst grade			
3	2 (66.7)	11 (50.0)	13 (52.0)
4	1 (33.3)	10 (45.5)	11 (44.0)
Any serious AE	2 (66.7)	8 (36.4)	10 (40.0)
AE leading to dose reduction	2 (66.7)	4 (18.2)	6 (24.0)
AE leading to dose interruption	3 (100.0)	18 (81.8)	21 (84.0)
AE leading to permanent discontinuation	1 (33.3)	8 (36.4)	9 (36.0)
Any ADR	3 (100.0)	22 (100.0)	25 (100.0)
ADR by worst grade			
3	2 (66.7)	13 (59.1)	15 (60.0)
4	1 (33.3)	6 (27.3)	7 (28.0)
Any serious ADR	2 (66.7)	5 (22.7)	7 (28.0)
ADR leading to dose reduction	2 (66.7)	4 (18.2)	6 (24.0)
ADR leading to dose interruption	3 (100.0)	18 (81.8)	21 (84.0)
ADR leading to permanent discontinuation	1 (33.3)	7 (31.8)	8 (32.0)

*ADR* adverse drug reaction, *AE* adverse event

**Table 4:** Treatment-emergent adverse events occurring in ≥ 20% of patients in the safety analysis set

TEAE, *n* (%) MedDRA system organ class MedDRA preferred term	Copanlisib		
	45 mg (*n* = 3)	60 mg (*n* = 22)	Total (*n* = 25)
Any TEAE	3 (100.0)	22 (100.0)	25 (100.0)
Metabolism and nutrition disorders	3 (100.0)	22 (100.0)	25 (100.0)
Hyperglycemia	3 (100.0)	19 (86.4)	22 (88.0)
Gastrointestinal disorders	3 (100.0)	19 (86.4)	22 (88.0)
Stomatitis	1 (33.3)	12 (54.4)	13 (52.0)
Diarrhea	1 (33.3)	9 (40.9)	10 (40.0)
Nausea	2 (66.7)	4 (18.2)	6 (24.0)
Constipation	0	5 (22.7)	5 (20.0)
Investigations	3 (100.0)	19 (86.4)	22 (88.0)
Neutrophil count decreased	2 (66.7)	13 (59.1)	15 (60.0)
WBC count decreased	3 (100.0)	11 (50.0)	14 (56.0)
Platelet count decreased	1 (33.3)	7 (31.8)	8 (32.0)
Lymphocyte count decreased	2 (66.7)	4 (18.2)	6 (24.0)
Infections and infestations	2 (66.7)	16 (72.7)	18 (72.0)
Nasopharyngitis	1 (33.3)	5 (22.7)	6 (24.0)
Blood and lymphatic system disorders	2 (66.7)	13 (59.1)	15 (60.0)
Anemia	1 (33.3)	6 (27.3)	7 (28.0)
Neutropenia	0	5 (22.7)	5 (20.0)
Vascular disorders	3 (100.0)	11 (50.0)	14 (56.0)
Hypertension	3 (100.0)	10 (45.5)	13 (52.0)
General disorders and administration site conditions	3 (100.0)	8 (36.4)	11 (44.0)
Pyrexia	1 (33.3)	5 (22.7)	6 (24.0)
Fatigue	2 (66.7)	3 (13.6)	5 (20.0)
Nervous system disorders	0	9 (40.9)	9 (36.0)
Dysguesia	0	7 (31.8)	7 (28.0)
Skin and subcutaneous tissue disorders	0	9 (40.9)	9 (36.0)
Rash	0	7 (31.8)	7 (28.0)
Psychiatric disorders	1 (33.3)	6 (27.3)	7 (28.0)
Insomnia	1 (33.3)	5 (22.7)	6 (24.0)
Eye disorders	0	6 (27.3)	6 (24.0)
Musculoskeletal and connective tissue disorders	0	5 (22.7)	5 (20.0)
Respiratory, thoracic and mediastinal disorders	1 (33.3)	4 (18.2)	5 (20.0)

*MedDRA* Medical Dictionary for Regulatory Activities, *TEAEs* treatment-emergent adverse events, *WBC* white blood cell

**Table 5:** Adverse drug reactions occurring in ≥ 10% of patients receiving copanlisib (either dose) in the safety analysis set

ADRs defined by MedDRA preferred term, *n* (%)	Copanlisib (*n* = 25)
Any ADR	25 (100.0)
Hyperglycemia	22 (88.0)
Neutrophil count decreased	14 (56.0)
Stomatitis	13 (52.0)
WBC count decreased	13 (52.0)
Hypertension	13 (52.0)
Diarrhea	7 (28.0)
Platelet count decreased	7 (28.0)
Dysgeusia	7 (28.0)
Nausea	6 (24.0)
Anemia	5 (20.0)
Neutropenia	5 (20.0)
Fatigue	5 (20.0)
Lymphocyte count decreased	5 (20.0)
Pyrexia	4 (16.0)
Rash	4 (16.0)
Thrombocytopenia	3 (12.0)
Herpes zoster	3 (12.0)
Weight decreased	3 (12.0)
Glucose tolerance impaired	3 (12.0)
Hypokalemia	3 (12.0)

*ADRs* adverse drug reactions, *MedDRA* Medical Dictionary for Regulatory Activities, *WBC* white blood cell

The most common types of AEs after 1 year of treatment initiation (*n* = 9) were hypertension (*n* = 5; 55.6%) and neutrophil count decreased (*n* = 4; 44.4%), followed by platelet count decreased, nasopharyngitis, and anemia (each *n* = 3; 33.3%). Notably, hyperglycemia occurred in two out of the nine patients (22.2%) (Supplementary Table S2).

No DLTs occurred at the 45 or 60 mg dose, so the recommended copanlisib dose for Japanese patients was determined to be 60 mg. Similarly, no DLTs were recorded during the expansion part. Twenty patients (80.0%) had a dose modification and 6(24.0%) required a dose reduction because of an AE (two in the 45 mg cohort [66.7%] and four in the 60 mg cohort [18.2%]). The events leading to dose reduction were all ADRs, of which hyperglycemia occurred in two patients (66.7%) in the 45 mg cohort and 1(4.5%) in the 60 mg cohort. Other events included neutropenia, febrile neutropenia, platelet count decreased, hypertension, and stomatitis in one patient each in the 60 mg cohort. In the three patients who required dose reduction due to cytopenia (i.e., neutropenia, febrile neutropenia, or platelet count decreased), the number of prior treatment lines was above the median (3.0 lines) in all three patients (10, 8, and 4 lines, respectively); therefore, as a trend, the number of prior treatment lines was higher when the dose reduction was caused by cytopenia. However, no consistent trend was observed between due to cytopenia and body surface area, which ranged from 1.34 to 1.89 m^2^ in these three patients.

Nine patients (36.0%) permanently discontinued copanlisib because of AEs, specifically because of arthritis (grade 1), cellulitis (grade 2), decreased appetite (grade 3), hypertension (grade 3), neutropenia (grade 2), neutrophil count decreased (grade 2), pneumonitis (grade 3), post-procedural infection (grade 2), Stevens-Johnson syndrome (grade 3), and stomatitis (grade 2) (each *n* = 1). Except for the arthritis, all these events were considered to be drug-related.

### Efficacy

**Fig. 2:**
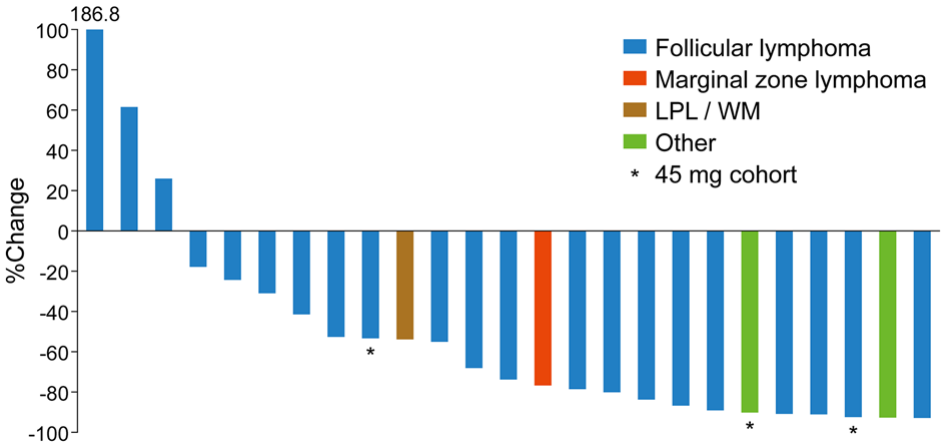
Waterfall plot showing the change in lesion size during treatment with copanlisib (*n* = 25). *LPL* lymphoplasmacytoid lymphoma, *WM* Waldenström macroglobulinemia

**Fig. 3:**
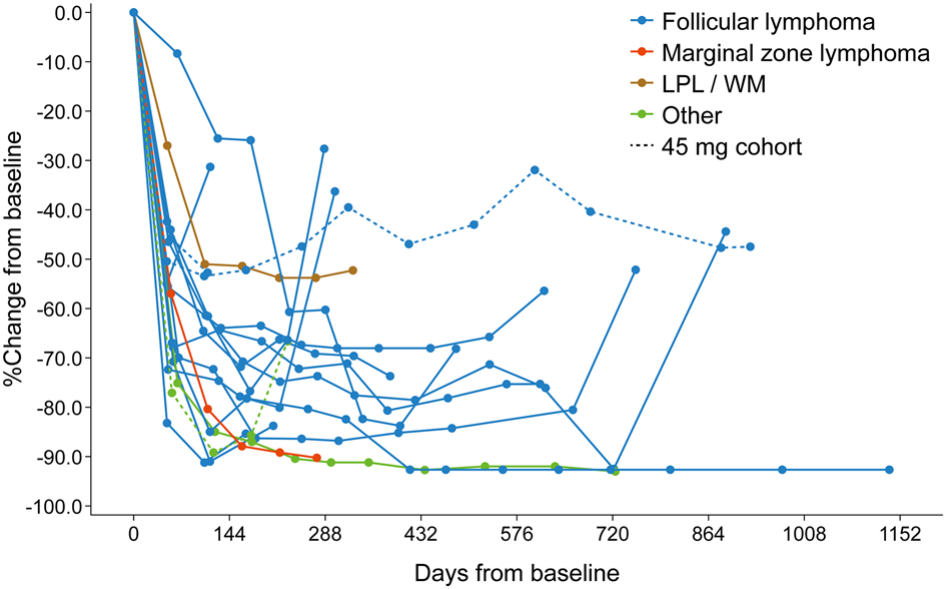
Spider plot showing the change from baseline in lesion size for each individual patient with a partial or complete response (*n* = 18). *LPL* lymphoplasmacytoid lymphoma, *WM* Waldenström macroglobulinemia

**Fig. 4:**
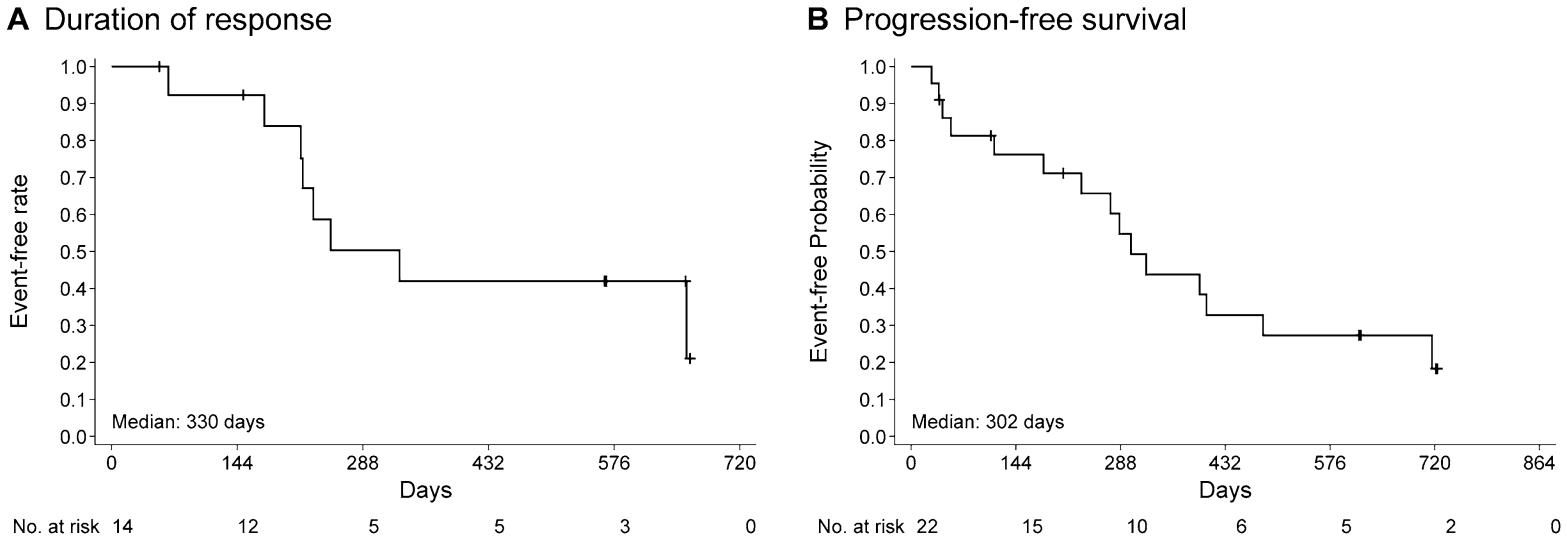
Kaplan–Meier analysis of (**A**) duration of response and (**B**) progression-free survival during treatment with copanlisib in the 60 mg dose cohort (*n* = 22)

Five patients had achieved CR (1 at copanlisib 45 mg and 4 at 60 mg), and 12 had achieved PR (2 at 45 mg and 10 at 60 mg), for an ORR of 68.0% (17/25 patients) (Table 6). An additional four patients in the 60 mg group (18.2%) had stable disease and three patients (13.6%) had PD. A reduction in lesion size was seen in 22/25 patients (88.0%), and 18/25 patients (72.0%) had a reduction in size of ≥ 50%. The best change from baseline in target lesion size is shown in Fig. 2, and the change from baseline in target lesion size for each treated patient with a response of PR or better is shown in Fig. 3. The median duration of response was 330 days (range 65–659 days; Supplementary Table S3 and Fig. 4A). Median PFS in the total cohort was 302 (95% CI 231, 484) days (Supplementary Table S4 and Fig. 4B).

**Table 6:** Antitumor response

	Response rate, *n* (%) [95% CI]		
	45 mg (*n* = 3)	60 mg (*n* = 22)	Total (*n* = 25)
Best response			
CR	1 (33.3) [0.84, 90.57]	4 (18.2) [5.19, 40.28]	5 (20.0) [6.83, 40.70]
PR	2 (66.7) [9.43, 99.16]	10 (45.5) [24.39, 67.79]	12 (48.0) [27.80, 68.69]
Stable disease	0 [0.00, 70.76]	4 (18.2) [5.19, 40.28]	4 (16.0) [4.54, 36.08]
Progressive disease	0 [0.00, 70.76]	3 (13.6) [2.91, 34.91]	3 (12.0) [2.55, 31.22]
Unconfirmed early stable disease^a^	0 [0.00, 70.76]	1 (4.6) [0.12, 22.84]	1 (4.0) [0.10, 20.35]
ORR (CR + PR + stable disease)	3 (100.0) [29.24, 100.00]	14 (63.6) [40.66, 82.80]	17 (68.0) [46.50, 85.05]
DCR (CR + PR)	3 (100.0) [29.24, 100.00]	17 (72.3)^b^ [54.63, 92.18]	20 (80.0) [59.30, 93.17]

*CI* confidence interval, *CR* complete response, *DCR* disease control rate, *ORR* objective response rate, *PR* partial response

^a^Assessment of stable disease earlier than 7 weeks after the start of treatment, with no radiologic assessment of stable disease, PR or CR at later cycles

^b^One patient with unconfirmed early stable disease in the 60 mg cohort was excluded from the DCR calculations

The median OS was not reached during the observation period because 6 of the 25 patients (24.0%) had died (2 in the 45-mg cohort and 4 in the 60 mg cohort) (Supplementary Table S5 and Supplementary Fig. S2). The 6-, 12-, and 24-month OS rates were 96.0, 92.0, and 84.0%, respectively (Supplementary Table S5).

### Pharmacokinetics

The maximum plasma concentration and area under the concentration–time curve to 168 h from copanlisib infusion in Japanese patients were both similar to those values determined in non-Japanese patients (Supplementary Fig. S3).

## Discussion

This phase Ib/II study demonstrated that the recommended dose of copanlisib is 60 mg in Japanese patients with relapsed/refractory indolent NHL, and that the safety and tolerability of copanlisib, as well as the antitumor activity, in this population are similar to those reported in the CHRONOS-1 study [13, 14]. No DLTs were reported and the recommended dose in Japanese patients (60 mg) is the same as the US-approved dose [9].

In our study, copanlisib was associated with an ORR of 68.0% and median duration of response of almost 1 year (330 days or 10.8 months), compared with an ORR of 60.6% and median duration of response of 14.1 months in CHRONOS-1 [14]. A high proportion of patients in our study (88.0%) showed a reduction in lesion size during treatment with copanlisib, which is similar to the 92.1% of patients in CHRONOS-1 who had a reduction in lesion size [14]. In addition, 72.0% of treated patients in our study showed a reduction in lesion size of ≥ 50% compared with 62.7% in CHRONOS-1 [14]. While this difference may seem somewhat marked, our study was small and there is no reason to consider that the difference is anything other than an artifact of statistical variation. The 2-year OS rate in CHRONOS-1 was 69.0% [14], whereas it was 84.0% in the current study.

Hyperglycemia and hypertension were common AEs with copanlisib in our study, consistent with previous data for copanlisib monotherapy [11, 14, 15] and recent data from the CHRONOS-3 study, in which copanlisib was combined with rituximab in patients with indolent NHL [16]. However, the incidence of hyperglycemia in our study (88.0%) was higher than in the CHRONOS-1 study (50.0%) [14]. The reason for the higher incidence of hyperglycemia in our study is not known. A similarly high incidence of hyperglycemia (80.0%) was reported in a phase I study of copanlisib in Japanese patients with advanced or refractory solid tumors [10]. The high incidence of hyperglycemia may be attributed to a possible increase in blood glucose after copanlisib administration, even in patients with borderline diabetes. In our study, hyperglycemia was transient during treatment with copanlisib, and blood glucose levels returned to normal at the end of treatment, regardless of baseline values. When needed, blood glucose levels were controlled with hyperglycemia medications. Hyperglycemia during copanlisib is caused by inhibition of PI3Kα, which is involved in insulin-like growth factor signaling [17]. It is generally asymptomatic and can usually be managed with intravenous or oral fluids. Copanlisib discontinuation is not necessary in patients who develop acute hyperglycemia [18], and none of the patients in our study discontinued treatment because of hyperglycemia.

Of the five patients with hypertension who were able to continue copanlisib for more than 1 year, three patients developed hypertension within the first month of treatment (grade 3, *n* = 2; grade 1, *n* = 1). Therefore, hypertension can be controlled by careful observation for 1 month after the start of treatment. The other two patients developed grade 2 hypertension 14 months and 15 months after the start of treatment. Further data on this AE are needed, since it appears that hypertension develops over time. No unexpected AEs developed in our study; all AEs were consistent with the known safety profile of copanlisib. In addition, drug exposure in Japanese patients was equivalent to that observed in non-Japanese individuals.

Our study has some limitations which need to be acknowledged. As a phase Ib/II study, a small number of patients were enrolled. Almost all of these patients had FL, so the results should be confirmed in larger studies in patients with other types of indolent NHL.

In conclusion, this study indicates that copanlisib, at a recommended dose of 60 mg, has an acceptable safety and tolerability profile, and showed promising antitumor activity in Japanese patients with relapsed/refractory indolent NHL.

## Supplementary Information

Below is the link to the electronic supplementary material.

Supplementary file1 (DOCX 239 KB)
