# Supplementary material for: Safety and antitumor activity of copanlisib in Japanese patients with relapsed/refractory indolent non-Hodgkin lymphoma: a phase Ib/II study
Source: Int J Hematol. 2022 Sep 29;117(1):100–9. doi: 10.1007/s12185-022-03455-0 (PMC9813077; doi:10.1007/s12185-022-03455-0)
Supplement: Supplementary file 1 — Supplementary file1 (DOCX 239 KB) [file 12185_2022_3455_MOESM1_ESM.docx]

**Supplementary materials for “The safety and antitumor activity of copanlisib in Japanese patients with relapsed/refractory indolent non-Hodgkin lymphoma: a phase Ib/II study”**

## Supplementary Methods

### Pharmacokinetic study

In the dose-escalation part of the study, blood samples were collected for pharmacokinetic analysis at the following times: pre-infusion and 5 minutes, 1, 1.5, 2.5, 5, 8, 24, 48–72, and 168 hours after start of the infusion on day 1 of cycle 1 (i.e., samples after 8 hours were taken on days 2, 3, and 8 of cycle 1); pre-infusion, and 5–15 minutes, 55 minutes, and 1.5–5 hours after the start of the infusion on day 15 of cycle 1.

In the dose expansion part of the study, blood samples were collected for pharmacokinetic analysis at the following times: pre-infusion and 5 minutes, 1, 1.5, 2.5, 5, and 8 hours after start of the infusion on day 1 of cycle 1; on day 8 of cycle 1 (168 after the day 1 infusion); and pre-infusion, and 5–15 minutes, 55 minutes and 1.5–5 hours after the start of the infusion on day 15 of cycle 1.

In both parts of the study, pharmacokinetic sampling could be undertaken in cycle 2 if it was not feasible to collect the samples during cycle 1. All the samples were stored at −70°C and transported on dry ice. Pharmacokinetic analyses were performed by NorthEast BioAnalytical Laboratories using liquid chromatography tandem mass spectrometry (LC-MS/MS).

The pharmacokinetic samples were analyzed for levels of copanlisib, its metabolite M-1, and other metabolites as needed.

Copanlisib plasma concentration data were used to estimate the individual maximum plasma concentration (C_max_) and the area under the concentration-time curve to 168 hours after infusion (AUC_0–168_).

A population pharmacokinetic model was developed to predict the copanlisib plasma concentration measurements of the patients in the study. To infer individual pharmacokinetic parameter estimates, empirical Bayes estimates were generated using the importance sampling methodology (estimation command: EONLY=1) in NONMEM version 7.4.3. The model evaluation, simulations, and all other graphical and statistical analyses of the data were undertaken using R software. Model fits were visually assessed using standard goodness of fit graphs.

Data on the pharmacokinetics of copanlisib after the third 60-mg dose in Japanese patients were compared with the equivalent data from previous studies conducted in non-Japanese patients (Bayer data on file).

## Supplementary Table S1.

Blood glucose levels at baseline and medication for hyperglycemia

| **Copanlisib dose** | **Patient** | **Gender** | **Age, years** | **Medication for hyperglycemia** | | |
| --- | --- | --- | --- | --- | --- | --- |
|  |  |  |  | **HbA1c, %^a^ Baseline** | **Medicine name** | **Start relative**  **to treatment** |
| 45 mg | 1^b^ | Male | 65 | 5.3 | Glactiv 50 mg QD  Humulin R PRN^c^  Lantus 6 IU QD | After  After  After |
|  | 2 | Female | 71 | **6.6** | Humulin R 4 and 6 units QD/BID; 8 units QD  Metgluco 250 mg TID | After  During |
|  | 3^d^ | Female | 71 | 5.8 | Metgluco 250 mg TID  Metgluco 500 mg TID  Lantus 4 and 8 units QD  Lantus 4 and 6 units QD | Before and during  During  During  After |
| 60 mg | 4 | Female | 73 | 5.9 | Glactiv 50 mg QD  Glactiv 100 mg QD  Metgluco 500 mg BID | During and after  After  After |
|  | 5 | Male | 67 | **6.3** | Apidra 4 units once  Glactiv 50 mg QD  Humulin R 2 IU/day; 2–6 IU/day; 8–12 IU/day; 4–16 IU/day; 2 units once  Metgluco 250 mg and 500 mg TID  Lantus 2 IU QD | After  During  After  During  After |
|  | 6 | Female | 75 | 5.5 | No treatment | - |
|  | 7 | Male | 73 | 5.9 | No treatment | - |
|  | 8 | Male | 51 | 5.6 | No treatment | - |
|  | 9 | Male | 66 | 5.6 | No treatment | - |
|  | 10 | Male | 39 | 5.2 | No treatment | - |
|  | 11 | Female | 75 | **6.2** | Novorapid 2 and 5 units BID/TID; 4 and 6 units QD/BID/TID  Tresiba 3 and 5 units QD | After  After |
|  | 12 | Female | 74 | 5.8 | No treatment | - |
|  | 13 | Male | 62 | 5.5 | Metgluco 250 mg BID | During |
|  | 14 | Male | 67 | 5.5 | No treatment | - |
|  | 15 | Female | 67 | 5.8 | No treatment | - |
|  | 16^b^ | Male | 61 | 5.5 | Glufast 10 mg TID | During |
|  | 17 | Male | 58 | 5.3 | No treatment | - |
|  | 18 | Female | 58 | 4.7 | No treatment | - |
|  | 19 | Female | 73 | 5.4 | No treatment | - |
|  | 20^d^ | Female | 68 | **6.2** | Glactiv 50 mg QD  Glactiv 100 mg QD  Farxiga 5 mg QD | Before, during, and after  During  During |
|  | 21 | Male | 65 | **6.1** | Insulin Aspart 1–7 units QD/BID/TID  Insulin Glargine 2–4 units QD  Sitagliptin phosphate monohydrate 25 mg and 50 mg QD  Tresiba FlexTouch 2, 6, and 10 units QD | After  After  During  During |
|  | 22 | Male | 60 | 5.4 | No treatment | - |
|  | 23 | Female | 58 | 5.6 | No treatment | - |
|  | 24 | Male | 68 | 5.2 | No treatment | - |
|  | 25 | Female | 57 | 5.2 | No treatment | - |

^a^HbA1c ≤6.0% is normal and ≥6.0% is high blood glucose (indicated in bold).

^b^Developed hyperglycemia within and after one year of treatment.

^c^Pro re nata (PRN) refers to the administration of prescribed medication whose timing is left to the patient.

^d^Two patients had received medication for diabetes mellitus before the start of treatment.

BID, two times a day; HbA1c, glycated hemoglobin, IU, international unit; QD, once a day; TID, three times a day.

## Supplementary Table S2.

Adverse events occurring in the year after copanlisib treatment initiation

| **TEAE, n (%)**  MedDRA preferred term | **Copanlisib**  **(*n* = 9)** | **AEs** | | | |
| --- | --- | --- | --- | --- | --- |
|  |  | **Persistent/ occasional^a^** | **Grade^b^** | **New^c^** | **Grade^b^** |
| Hypertension | 5 (55.6) | 3 (33.3) | 3, 3, 2 | 2 (22.2) | 2, 2 |
| Neutrophil count decreased | 4 (44.4) | 3 (33.3) | 4, 3, 3 | 1 (11.1) | 3 |
| Platelet count decreased | 3 (33.3) | 3 (33.3) | 2, 2, 2 | - | - |
| Nasopharyngitis | 3 (33.3) | 2 (22.2) | 2, 1 | 1 (11.1) | 2 |
| Anemia | 3 (33.3) | 2 (22.2) | 2, 1 | 1 (11.1) | 2 |
| Hyperglycemia | 2 (22.2) | 2 (22.2) | 3, 3 | - | - |
| Diarrhea | 2 (22.2) | 1 (11.1) | 1 | 1 (11.1) | 1 |
| Sinusitis/acute sinusitis | 2 (22.2) | - | - | 2 (22.2) | 3, 2 |
| Lipase increased | 2 (11.1) | 2 (22.2) | 1, 1 | - |  |
| Pharyngitis | 1 (11.1) | 1 (11.1) | 1 | - |  |
| Laryngitis | 1 (11.1) | 1 (11.1) | 1 | - |  |
| WBC count decreased | 1 (11.1) | 1 (11.1) | 2 | - |  |
| ALT increased | 1 (11.1) | 1 (11.1) | 2 | - |  |
| AST increased | 1 (11.1) | 1 (11.1) | 1 | - |  |
| Gastritis | 1 (11.1) | - |  | 1 (11.1) | 1 |
| Constipation | 1 (11.1) | 1 (11.1) | 1 |  |  |
| Lymphocyte count increased | 1 (11.1) | - |  | 1 (11.1) | 2 |
| Lymphocyte count decreased | 1 (11.1) | 1 (11.1) | 2 |  |  |
| Hepatic function abnormal | 1 (11.1) | - |  | 1 (11.1) | 3 |
| Upper respiratory tract infection | 1 (11.1) | 1 (11.1) | 1 |  |  |
| Pneumonia | 1 (11.1) | 1 (11.1) | 2 |  |  |
| Retinal detachment | 1 (11.1) | - |  | 1 (11.1) | 3 |
| Retinal tear | 1 (11.1) | - |  | 1 (11.1) | 1 |
| Keratitis | 1 (11.1) | - |  | 1 (11.1) | 1 |
| Eyelid edema | 1 (11.1) | - |  | 1 (11.1) | 1 |
| Herpes zoster | 1 (11.1) | - |  | 1 (11.1) | 2 |
| Post herpetic neuralgia | 1 (11.1) | - |  | 1 (11.1) | 2 |
| Pyrexia | 1 (11.1) | 1 (11.1) | 1 |  |  |
| Insomnia | 1 (11.1) | - |  | 1 (11.1) | 1 |
| Delirium | 1 (11.1) | - |  | 1 (11.1) | 1 |
| Blood LDH increased | 1 (11.1) | 1 (11.1) | 1 |  |  |
| Arthritis | 1 (11.1) | 1 (11.1) | 2 |  |  |
| Colitis | 1 (11.1) | - |  | 1 (11.1) | 1 |
| Enterocolitis | 1 (11.1) | - |  | 1 (11.1) | 1 |
| Infusion-related reaction | 1 (11.1) | - |  | 1 (11.1) | 2 |
| Nausea | 1 (11.1) | - |  | 1 (11.1) | 1 |
| Infection | 1 (11.1) | - |  | 1 (11.1) | 2 |
| Weight decreased | 1 (11.1) | - |  | 1 (11.1) | 2 |
| Blood alkaline phosphatase increased | 1 (11.1) | - |  | 1 (11.1) | 1 |
| Hypoalbuminaemia | 1 (11.1) | - |  | 1 (11.1) | 2 |

^a^Persistent/occasional AEs are AEs that have also occurred within one year.

^b^Worst grade of the individual AE is shown.

^c^New AEs are AEs that occurred only after one year.

AE, adverse event; ALT, alanine aminotransferase; AST, aspartate aminotransferase; LDH, lactate dehydrogenase; MedDRA, Medical Dictionary for Regulatory Activities; TEAE, treatment-emergent adverse event; WBC, white blood cell.

## Supplementary Table S3.

Duration of treatment response (independent assessment)

|  | **Copanlisib** | | |
| --- | --- | --- | --- |
|  | **45 mg** | **60 mg** | **Total** |
| No. of patients in this analysis | 3 | 14 | 17 |
| With event, n (%) | 2 (66.7) | 8 (57.1) | 10 (58.8) |
| Censored, n (%) | 1 (33.3) | 6 (42.9) | 7 (41.2) |
| Duration of response, days |  |  |  |
| Median (95% CI) | 335.5 (147–497) | 330 (175–NE) | 330 (175–NE) |
| Range without censored values | 174–497 | 65–659 | 65–659 |

CI, confidence interval; NE, not estimable due to censored data.

## Supplementary Table S4.

Progression-free survival (independent assessment)

|  | **Copanlisib** | | |
| --- | --- | --- | --- |
|  | **45 mg (*n* = 3)** | **60 mg (*n* = 22)** | **Total (*n* = 25)** |
| Number (%) of patients |  |  |  |
| With event | 2 (66.7) | 15 (68.2) | 17 (68.0) |
| Censored | 1 (33.3) | 7 (31.8) | 8 (32.0) |
| PFS, days |  |  |  |
| Median (95% CI) | 416.5 (231–602) | 302 (182–484) | 302 (231–484) |
| Range without censored values | 231–602 | 28–716 | 28–716 |
| PFS rate, % (95% CI) |  |  |  |
| 6 months | 100.0 (100–100) | 71.0 (47–86) | 75.0 (52–88) |
| 12 months | 50.0 (1–91) | 44.0 (22–64) | 45.0 (23–64) |
| 18 months | 50.0 (1–91) | 27.0 (10–48) | 30.0 (12–50) |

CI, confidence interval; PFS, progression-free survival.

## Supplementary Table S5.

Overall survival (independent assessment)

|  | **Copanlisib** | | |
| --- | --- | --- | --- |
|  | **45 mg (*n* = 3)** | **60 mg (*n* = 22)** | **Total (*n* = 25)** |
| Number (%) of patients |  |  |  |
| With event | 2 (66.7) | 4 (18.2) | 6 (24.0) |
| Censored | 1 (33.3) | 18 (81.8) | 19 (76.0) |
| OS, days |  |  |  |
| Median (95% CI) | 1064 (498–NE) | NE (918–NE) | NE (918–NE) |
| Range without censored values | 498–1064 | 154–918 | 154–1064 |
| OS rate, % (95% CI) |  |  |  |
| 6 months | 100.0 (100–100) | 95.0 (72–99) | 96.0 (75–99) |
| 12 months | 100.0 (100–100) | 91.0 (68–98) | 92.0 (72–98) |
| 18 months | 67.0 (5–95) | 86.0 (63–95) | 84.0 (63–94) |
| 24 months | 67.0 (5–95) | 86.0 (63–95) | 84.0 (63–94) |

CI, confidence interval; NE, not estimable due to censored data; OS, overall survival.

## Supplementary Fig. S1

Change in blood glucose over time in cycle 1 in one patient with high baseline HbA1c (A) and one patient with normal baseline HbA1c (B). Hyperglycemia was defined as blood glucose ≥160 mg/dL and HbA1c >6.0%. The graphs correspond to patient 2 and patient 10 in supplementary table S5. C, cycle; D, day; HbA1c, glycated hemoglobin.


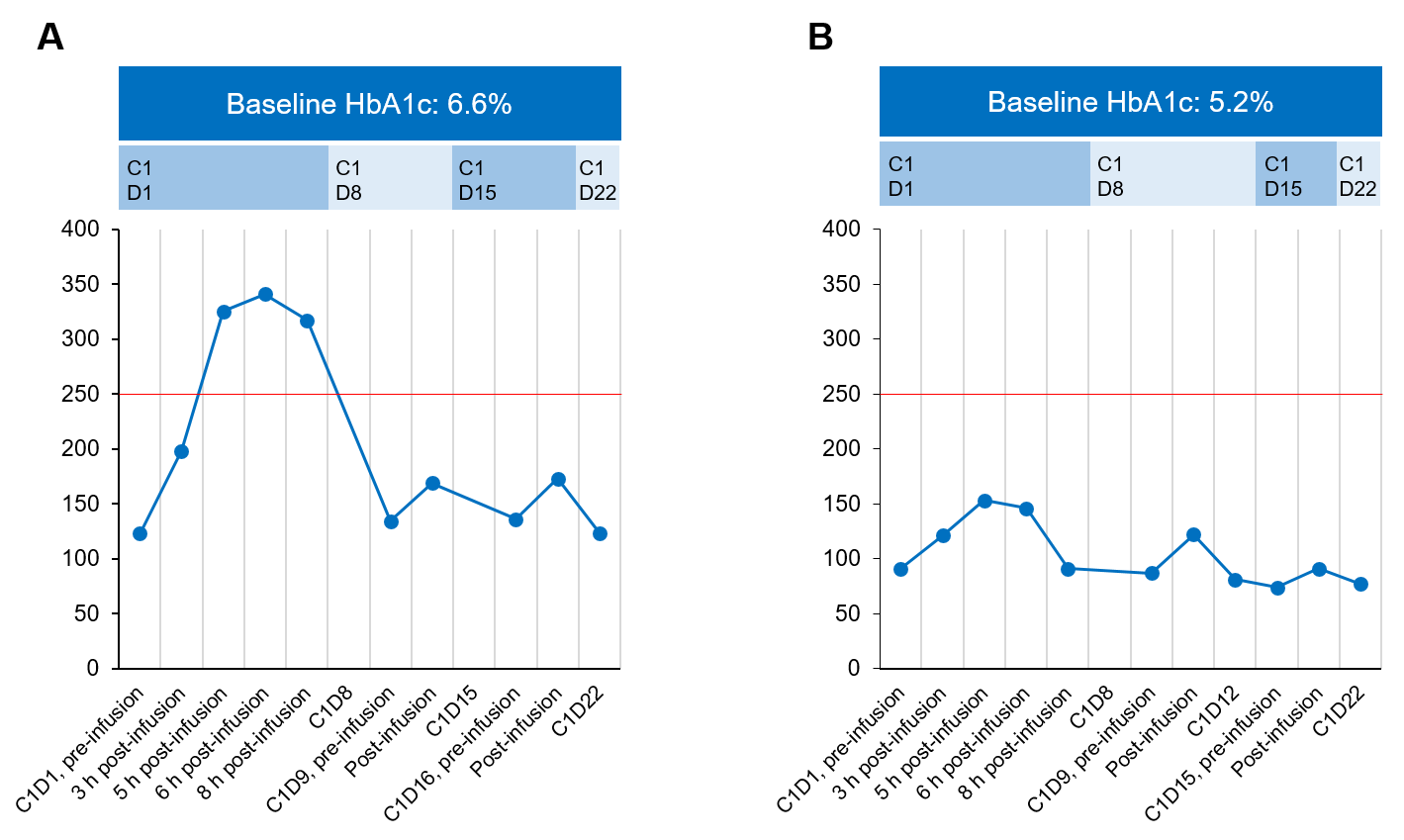


## Supplementary Fig. S2

Kaplan–Meier analysis of overall survival during treatment with copanlisib in the 60-mg dose cohort (n = 22).


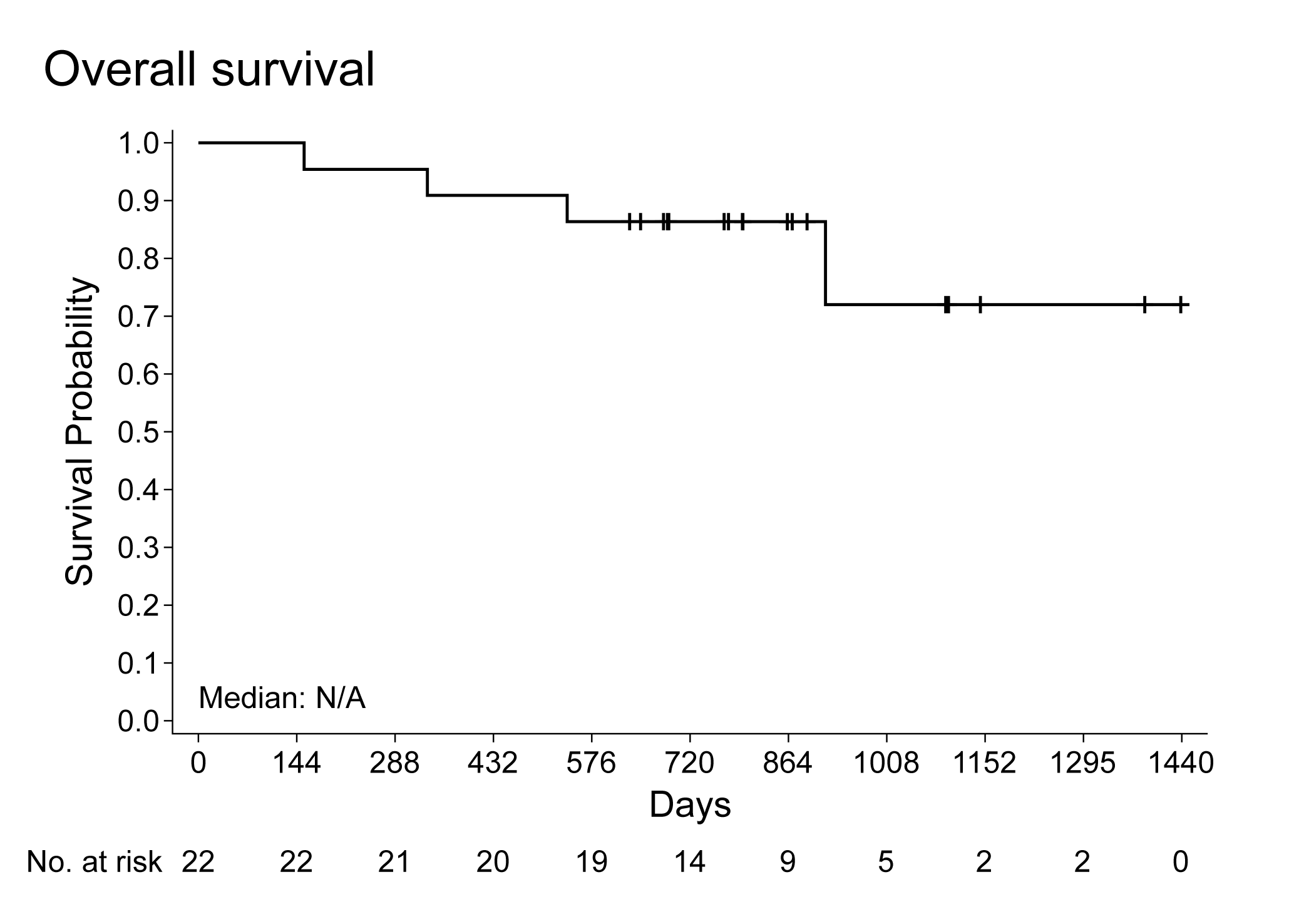


## Supplementary Fig. S3

Boxplots showing (A) maximal plasma copanlisib concentration (C_max_) and (B) area under the concentration-time curve from baseline to 168 hours after the third infusion (AUC_0–168_) of copanlisib 60 mg in Japanese patients compared with data from non-Japanese patients in previous studies (data on file). Data for Japanese patients are from the current study and a previous phase I study (study 15205) with copanlisib monotherapy. The box marks the range between the upper and lower quartiles (interquartile range), and the central black horizontal line indicates the median value. The upper and lower whiskers indicate data within 150% of the interquartile range of the upper and lower quartiles, respectively. Individual estimates are shown as open circles. ND, nominal doses.
